# Histamine and histamine receptors: Roles in major depressive disorder

**DOI:** 10.3389/fpsyt.2022.825591

**Published:** 2022-09-23

**Authors:** Hong Qian, Chang Shu, Ling Xiao, Gaohua Wang

**Affiliations:** ^1^Department of Psychiatry, Renmin Hospital of Wuhan University, Wuhan, China; ^2^Division of Child Healthcare, Department of Pediatrics, Tongji Hospital, Tongji Medical College, Huazhong University of Science and Technology, Wuhan, China

**Keywords:** histamine, histamine receptors, immune regulation, major depressive disorder, therapy

## Abstract

Although the incidence of major depressive disorder (MDD) is high and its social impact is great, we still know very little about the pathophysiology of depression. The monoamine hypothesis of depression suggests that 5-HT, NE, and DA synergistically affect mood, which is the basis of current drug therapy for depression. However, histamine as a monoamine transmitter is rarely studied. Our review is the first time to illustrate the effect of histaminergic system on depression in order to find the way for the development of new antidepressant drugs. The brain neurotransmitter histamine is involved in MDD, and the brain histaminergic system operates through four receptors. Histamine and its receptors can also regulate the immune response to improve symptoms of depression. In addition, H3R can interact with other depression-related transmitters (including 5-HT, DA, GLU, and MCH); thus, histamine may participate in the occurrence of depression through other neural circuits. Notably, in rodent studies, several H3R and H1R antagonists were found to be safe and effective in alleviating depression-like behavior. To highlight the complex functions of histamine in depression, and reveals that histamine receptors can be used as new targets for antidepressant therapy.

## Introduction

Major depressive disorder (MDD) is a common and serious illness that affected more than 200 million people in 2017 and is estimated to have the second highest global disease burden by 2020 (1). Many factors, including heredity, neurotransmitters, immunity, oxidation, and the inflammatory system, are involved in the pathophysiology of MDD (2, 3). However, the pathological mechanism of MDD is not yet clear. In addition, current treatments for depression do not effectively or adequately reduce the associated morbidity and mortality. In fact, as many as 50% of MDD patients do not experience complete remission after receiving antidepressant medication (4). These data highlight the importance of depressive disorder as a priority for public health projects and the importance of effective interventions to alleviate this burden.

Neuromodulation system dysfunction is common in depression. Based on the monoamine hypothesis of depression, monoamine reuptake inhibitors have been developed into antidepressants and have recently been widely used in the clinic (5). The relationship among serotonin, norepinephrine and dopamine (DA) in depression has been extensively studied, but the role of histamine, which is also a monoamine transmitter, is poorly understood (6). Histamine and its receptors were originally described as part of the immune and gastrointestinal systems, but their presence in the central nervous system (CNS) and importance in behavior are receiving increasing attention in CNS diseases, such as Alzheimer’s disease, depression, sleep disorders, drug dependence disorders, and Parkinson’s disease (6).

All effects of histamine are regulated by the G protein-coupled receptors the H1 receptor (H1R), H2R, H3R, and H4R (7). H1R, H2R, and H3R can be found in the CNS, where they act to regulate multiple physiological functions, such as energy homeostasis, cognition and attention, sensory and motor functions, and so on (8). Moreover, two of the histamine receptors are used in the clinical treatment of mental diseases. H1R is related to sleep, and H1R antagonists can be used to treat chronic insomnia (9). H3R is involved in cognition and attention, and H3R agonists can be used to treat alcohol addiction (10), Alzheimer’s disease (11) and narcolepsy (12). To date, few studies have addressed the relationship between histamine and depression, and this direct relationship is unclear. This review discusses the role and involvement of histamine in MDD, with a focus on histamine receptors, and includes an examination of immune responses and the interaction between histamine and other transmitters. In addition, this review highlights the activity of histamine receptor agonists and antagonists as potential antidepressants in basic and preclinical studies. This review focuses primarily on recently published data; however, data from early studies are also offered to highlight the various developmental stages of the histamine theory in depression.

## The neurotransmitters related to depression

Stress plays a fundamental role in many causes of depression. Stress activates the HPA axis and causes the hypothalamus to release corticotropin-releasing hormone, which acts on the anterior pituitary and releases adrenocorticotropic hormone and finally triggers the adrenal cortex to release glucocorticoid and monoamine transmitters. MDD is associated with the deficiency of monoamines, especially 5-hydroxytryptamine (5-HT), noradrenaline (NA), and dopamine (DA), in the brain (13). The treatment of affective disorders is primarily based on the enhancement of the noradrenergic and serotonergic systems by selective or non-selective reuptake inhibitors.

In the dopaminergic system, impaired neurotransmission is also associated with depression, and some preclinical studies have shown that agonists of dopaminergic receptors (D1R, D2R, and D3R, respectively) produce an antidepressant effect (14). Moreover, pure dopaminergic drugs such as pramipexole, DA precursors and DA reuptake inhibitors have been shown to have a therapeutic effect on depression (15).

Importantly, in addition to monoamines, other neurotransmitters, such as the glutamatergic or gamma-aminobutyric acid (GABA)ergic melanin-concentrating hormone (MCH), are involved in the pathophysiology of depression (16–20). Abnormal glutamatergic neurotransmission is involved in the development of mental illnesses, including schizophrenia, bipolar disorder and depression (16). Glutamate (GLU) receptors are classified as N-methyl-D-aspartate (NMDA), alpha-amino-3-hydroxy-5-methyl-4-isoxazolepropionic acid (AMPA) and metabotropic receptors (16). Among these classes, the NMDA receptor (NMDAR) is considered to be the main target for the development of a new generation of antidepressant drugs (16, 17). In fact, NMDAR antagonists such as ketamine can alleviate depressive symptoms in patients within hours of administration (18, 19). MCH is a 19-amino-acid cyclic peptide whose biological function is mediated by two G protein-coupled receptors called MCH receptor 1 (MCHR-1) and MCHR-2 (20). Preclinical studies suggest that the MCHergic system is involved in depression. Intra-locus coeruleus (LC) and intracerebroventricular injections of MCH produce depressive-like behavior, but this effect can be blocked by pretreatment with the MCH-R1 antagonist SNAP-94847 in the LC (21). In a clinical study, MCH serum levels were significantly decreased in patients with MDD after 4 weeks of antidepressant treatment (22). Overall, preclinical studies have demonstrated that antagonism of the MCHergic system may be a new treatment approach for antidepressant drugs.

Monoamine transmitters such as 5-HT, NA, and DA and non-monoamine transmitters such as GABA, NMDA, and MCH have been shown to be associated with the pathophysiology of depression. As a monoamine transmitter, histamine can interact with these transmitters and may also play an important role in depression, but research on this role is scarce.

## The function of histamine and the histamine receptor in the brain

Histamine plays an important role as a neurotransmitter in the brain, and its receptors have been shown to regulate many physiological functions that are affected in numerous CNS diseases (7, 8, 23) (Table 1).

**TABLE 1:** Overview of histamine receptors in central nervous system (CNS).

	H1R	H2R	H3R	H4R
G protein coupling	Gaq	Gas	Gai/o	Gai/o
Signal	Activate PLC, PKC, Ser/Thr and calcium release (25)	Increase cAMP and activates PKA (25, 28)	Activate MAPK, PKB and calcium release (36)	Activate MAPK, PKB, calcium release and (7, 42)
Location	CNS, lungs, and blood vessels (24)	Stomach, CNS and heart (7, 24)	CNS (37)	Immune cells (eosinophils, mast cells and dendritic cells) (7, 40)
CNS areas	Brain stem, thalamus, hypothalamus, septum, hippocampus, olfactory bulb amygdala and cortex (26).	Basal ganglia, hippocampus, amygdala, and cerebral cortex (29, 30).	Cerebral cortex, striatum, and hippocampus (29, 33, 34)	Not exist or amygdala, thalamus, cortex, hippocampus, amygdala and spinal cord (41, 42)
CNS function	Motor function, mood, arousal, sleep, cognitive function, pain perception, circadian rhythm, food intake thermoregulation and energy consumption (24, 26)	Circadian rhythm, cognitive process, food intake and glucose metabolism (24)	Pain perception, locomotor activity, circadian rhythm, memory, anxiety, food intake and cognition (38)	Immune function

The H1 receptor (H1R) is expressed in the CNS, lungs, and blood vessels (24). In the CNS, the main signaling pathway of H1R activates phospholipase C and protein kinase C (PKC) and catalyzes Ser/Thr phosphorylation of various downstream effectors, which subsequently induces intracellular Ca^2+^ release (25). H1R can activate most neurons in most brain regions, such as the brain stem, hypothalamus, hippocampus, thalamus, olfactory bulb, amygdala, septum, and cortex, by increasing Ca^2+^ (26). H1R participates in the regulation of motor function, mood, arousal, sleep and circadian rhythms, cognitive function, and pain perception (24). Moreover, H1R is involved in the modulation of several brain-controlled physiological functions, such as thermoregulation, food intake and energy consumption (24, 26). Blocking H1R with antagonists can increase the sensitivity of the CNS to seizures and sedation (24, 27). In addition, H1R antagonists can cross the blood–brain barrier (BBB) and cause drowsiness and are occasionally used to treat insomnia (9).

H2R is expressed in the stomach, CNS and heart (7, 24). Activation of H2R can stimulate the adenylyl cyclase, which in turn synthesizes cyclic adenosine monophosphate(cAMP). The cAMP then activates protein kinase A and the transcription factor cAMP response element-binding protein, which regulate neuronal physiology and plasticity (25, 28). In the human brain, H2R is widely distributed in the basal ganglia, hippocampus, amygdala, and cerebral cortex (29, 30). The function of H2R in the brain includes the modulation of circadian rhythm, cognitive processes, and food intake and glucose metabolism (24). Although H2R mediates important functions, and reports of CNS toxicity are infrequent, therapeutic applications for CNS-permeable ligands are relatively rare to date. Several studies have highlighted the potential of H2R antagonists to enhance the effects of opiate analgesics (31) and to exert a positive therapeutic effect on schizophrenia (32).

H3R is abundantly expressed in important areas of the brain, including the cerebral cortex, striatum, and hippocampus (29, 33, 34). H3R is characterized as an auto- and heteroreceptor that can regulate the synthesis and release of histamine or regulate the release of other neurotransmitters to influence the balance of different neurotransmitters (35, 36). Activation of Gi/o proteins by H3R results in the activation of mitogen-activated protein kinase (MAPK) pathways (36, 37). H3R regulates numerous behaviors, such as pain perception, locomotor activity, memory, food intake, circadian rhythms, anxiety, and cognition (38). Moreover, H3R antagonists can reduce alcohol intake in alcohol-preferring rats (10, 38), and inverse H3R agonists are used in clinical trials for the treatment of Alzheimer’s disease (11) and narcolepsy (12).

H4R was the last histamine receptor to be identified and is classified as a Gi/o-coupled GPCR (38, 39).H4R is mainly related to immune function and is expressed on immune cells, including eosinophils, mast cells and dendritic cells, where it induces calcium mobilization and chemotaxis (7, 40). However, the expression and function of H4R in the CNS remain controversial and require further research. In the human brain, H4R mRNA was detected in the amygdala, hippocampus, thalamus, cortex and spinal cord amygdala (41). By contrast, another report showed the opposite result, indicating that H4R may not exhibit functional expression on neurons in the CNS; thus, H4R may not be present on neurons in the brain but may be present on brain microglia, endothelial cells or epithelial cells (42).

H1R, H2R, and H3R are all expressed in the CNS and play physiological roles which are associated with depressive symptoms. The presence of H4R in the CNS remains controversial, but studies have linked it to immune function, and a growing body of research supports the immune inflammatory response to depression. Recent advances in our knowledge and understanding of histamine pharmacology, along with the identification and structural understanding of histamine receptors over the past decade, indicate the potential of new, improved antihistamines for future clinical development. Thus, there is still much to understand about the functional and pharmacological selectivity of these four receptors.

## The role of the histaminergic system in the pathophysiology of depression

### Histamine and the histamine receptor in brain areas relevant to depression

The histaminergic neurons in the mammalian brain are located in the tuberomammillary nucleus (TMN) of the posterior hypothalamus, which sends projection signals to all major parts of the brain (43), especially the areas important for cognitive function, such as the hippocampus, frontal cortex, basal forebrain and amygdala (44).

Brain histamine can confer biological protective effects against various unfavorable phenomena, such as denervation hypersensitivity, convulsions, stress susceptibility, ischemic injury and drug sensitization. A postmortem study of anterior cingulate cortex (ACC) and dorsolateral prefrontal cortex (DLPFC) tissues from individuals with depressive disorder bipolar disorder and control individuals evaluated the expression of histamine receptors and the enzymatic breakdown of histamine and histamine N-methyltransferase (HMT), indicating no change in the expression of these molecules except for a significant decrease in HMT mRNA expression in the ACC of individuals with MDD (45). In addition, a clinical study showed that the histamine level in 184 first-episode adolescent patients was significantly higher than that in the controls (46). The inconsistent results between peripheral and central histamine studies are due to different measurement methods and BBB barrier effects. The BBB is the primary barrier to the CNS and prevents the influx of active substances from the surrounding circulatory system. The BBB is closely associated with MDD and other mental disorders, such as Alzheimer’s and Parkinson’s diseases. Histamine has been shown to be a major factor leading to high BBB permeability (47). Histamines generally do not pass through the BBB; however, histamine induces an increase in BBB permeability, which in turn promotes histamine entry into the CNS. Previous reports have supported this view by suggesting that people with various neurodegenerative diseases have increased levels of histamine in their cerebrospinal fluid and brain parenchyma (47, 48). However these studies may indicate a change in histaminergic system of patients with depression, but more clinical researches are needed to confirm the role of histamine in the development and treatment of depression.

Not only histamine, but also histamine receptors are differentially expressed inpatients with depression. Positron emission tomography (PET) studies in humans have shown that histaminergic nervous system dysfunction, especially H1R dysfunction, is associated with depression (49). H1R binding in the prefrontal and frontal cortices and the cingulate gyrus was significantly decreased in patients with depression, and this reduction is associated with the severity of depression symptoms (50). H3R, which can regulate the release of histamine as an autoreceptor or regulate the release of other neurotransmitters as part of a heteroreceptor, is located on the dendrites and axons of histaminergic neurons (44). The H3 autoregulatory system seems to be linked to behavioral changes caused by stress, decreased sleep, inflammation, and oxidative stress. *In vitro* experiments have suggested that some of the beneficial effects of H3R antagonists on mood and cognition may be mediated through H4R (51). A recent study showed that H4R knockout mice had depression-like symptoms and cognitive impairment, but the study did not provide any mechanistic insights (52). A recent study provided the first description of the behavioral phenotype of H4R-deficient (H4R knockout) mice, which finally showed that H4R modulates various symptoms of depression, such as locomotor activity, anxiety, depression and feeding behavior (52). Both human and animal studies have found that the expression of histamine receptor is different between depressed patients and normal control subjects and between animal models of depression and related control animals, which is related to depression symptoms. Further animal studies have found that depression-like symptoms can be caused by knocking down the H4 receptor, and be improved by using an H3 receptor antagonist (51, 52).

Although none of depression’s symptoms are directly related to the histaminergic neuronal system, many brain regions show high levels of histamine and histamine receptors which can be used as indirect evidence that depression is related to histamine (45, 46, 49, 50).

### Interactions between histamine and other transmitters related to depression

**FIGURE 1:**
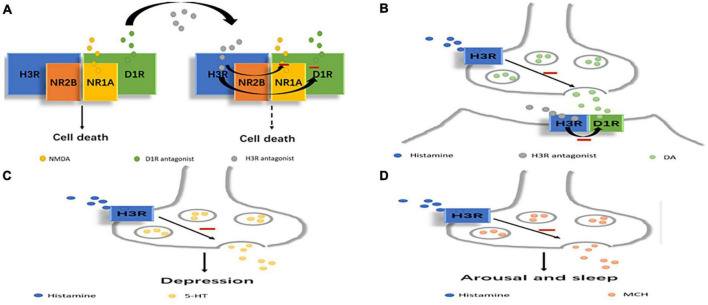
Scheme depicting how histamine may regulate other transmitters related to depression. **(A)** H3R antagonists can reduce NMDA or D1 receptor-mediated excitotoxic cell death by the D1R-H3R-NMDAR heterocomplex. **(B)** D1R-H3R heteromer can integrate DA- and histamine-related signaling which can be inhibited by H3R antagonists. **(C)** H3R can affect depression by directly inhibiting 5-HT release. **(D)** H3R can affect arousal and sleep by directly inhibiting MCH release.

Monoamine transmitters such as 5-hydroxytryptamine (5-HT), noradrenaline (NA), and dopamine (DA), and non-monoamine transmitters such as the GABA, N-methyl-D-aspartate (NMDA) and ergic MCH have been shown to be associated with the pathophysiology of depression (13, 16, 17, 21). As a monoamine transmitter, histamine interacts with these transmitter systems to form a network that mediates sleep-wake regulation, circadian and eating rhythms, immunity, learning, attention, motor activity, and memory (53, 54) (Figure 1).

Histamine released from brain mast cells may modulate the mood state *via* interactions with other transmitters, including serotonin and NA. Mast cell-deficient mice exhibited higher anxiety and depression levels than wild-type mice, and the symptoms were inhibited by injection of imipramine, an inhibitor of serotonin and NA uptake (55). H3R inhibits 5-HT release in the substantia nigra pars reticulata (SNr); this effect is attributed to its direct action on 5-HT terminals (56). Histamine acting through H3R tonically hyperpolarizes 5-HT neurons and inhibits 5-HT signaling, and when this inhibition is relieved by H3R antagonism, 5-HT neuron excitability is increased (57). The observed H3R-mediated endogenous control of 5-HT neurons provides even more compelling evidence that H3R is a candidate target for antidepressant drugs (57).

H3R antagonists have been shown to regulate the dopaminergic signaling pathway, and in the striatum, DA receptor agonists increase DA receptor signaling in wild-type mice but not in H3R knockout mice (34). A recent study investigated the existence of D1R-H3R heteromers, which constitute unique devices that can direct dopaminergic and histaminergic signaling toward the MAPK pathway in a Gs-independent and Gi-dependent manner (58). An imbalance between the histamine regulatory function (decreased) and DA (increased *via* D1R) was demonstrated in the striatum of mice with congenital hyperammonemia, providing a theoretical basis for the development of aminergic wake-promoting therapeutics to treat hyperammonemic disorders (59).The D1R-H3R heteromer is expressed in the brain, and it can selectively link GABAergic neurons and MAPK signals in the direct striatal pathway. These results indicate that the D1R-H3R heteromer can integrate DA- and histamine-related signaling involved in the control of the direct striatal pathway in striatal neurons (36). Moreover, the formation of heteromers by D1R, H3R, and NR1A-NR2B NMDARs allows close contact between D1R, H3R and the two NMDAR subunits. Structural changes in the entire macromolecular complex by H3R antagonists appear to reduce NMDA or D1 receptor-mediated excitotoxic cell death in cortical cultures, and the results indicate that H3R antagonists have neuroprotective potential, and H3R in the D1R-H3R-NMDAR heterocomplex is expected to be a target for preventing neurodegeneration (60).

Melanin-concentrating hormone neurons express H3R but not H1R or H2R (61). Histamine directly inhibits MCH neurons through H3R by activating G protein-dependent inwardly rectifying potassium (GIRK) channels (61). This observation strongly suggests that histamine and MCH cooperate in the regulation of arousal and sleep. In addition, histamine can inhibit MCH through H3R (61). MCH is associated with depression because injecting MCH into the dorsal raphe nucleus causes a depressive phenotype in rats, possibly because MCH inhibits serotonergic neurons, and MCHR-1 antagonists produce an antidepressant effect (62). Thus, high levels of histamine reduce sleep through the activation of H3R in the MCH system, and low levels of histamine may be associated with increased MCH activity and depressive behavior. A decrease in the histamine level may lead to depression by increasing MCH and histamine-derived functions such as physical activity, arousal, cognitive function, etc.

Since these H3R-expressing cells release many neurotransmitters regulated by H3R and the interactions among them, the role of H3R antagonists in depression may result from circuits involving many of these transmitters.

### Histamine regulates the immune system to ameliorate depression

Neuroimmune dysregulation is ubiquitous in different types of CNS diseases, and histamine is a pleiotropic monoamine that is involved in various neurophysiological functions and is a powerful regulator of the neuroimmune system (63, 64). An increasing amount of data have suggested that the immune system, including the cytokine network and innate immune response, plays a potential role in the etiology and pathophysiology of MDD (65–67). However, the detailed mechanism of immunity in depression has not been fully elucidated. A 6-year follow-up longitudinal study of 2,981 MDD patients (68) showed the associations of diagnosis and symptoms of depression with the interleukin-6 (IL-6) level, along with the ability of the IL-6 level to predict current MDD and greater symptom severity at follow-up. These results highlight the importance of investigating IL-6 as a potential marker. Twenty-five patients with unipolar MDD and 34 patients with schizophrenia, along with 50 healthy controls, were assessed for the presence of non-specific (auto-) immune antibodies, and significant differences were found, revealing the existence of a non-specific autoimmune disposition or reaction in at least a subgroup of patients with major depression and schizophrenia (69). Data from patients demonstrated a significant increase in peripheral urinary histamine levels in the group with depression compared with the control group, and the increased histamines further regulate the effects of inflammation (70).

Experiments with knockout mice revealed that H1R inhibits Th2 cell polarization, that H2R promotes Th1- and Th2-mediated immune responses, and that H4R affects innate and adaptive immunity *via* anti- or proinflammatory functions depending on the specific disease studied (40). In addition, H3R was involved in the inflammatory response only in the autoimmune encephalomyelitis model (71). Thus, the published studies highlight the complexity of histamine function in inflammation and the immune system.

Recently, all four histamine receptors have been shown to be expressed on immune cell microglia in the brain (72, 73). Histamine can stimulate microglial activation and the subsequent production of the proinflammatory factors tumor necrosis factor (TNF)-alpha and IL-6 *via* H1R and the H4R-MAPK and PI3K/AKT–NF-kappa B signaling pathways (72). Moreover, histamine induces microglial migration through H4R and inhibits lipopolysaccharide (LPS)-induced interleukin-1 beta (IL-1b) production (73). A selective and potent H3R inverse agonist suppressed microglial activation, chemotaxis and phagocytosis to ameliorate LPS-induced depression-like behavior in mice (74). Thus, histamine can indirectly affect neuronal function by influencing microglia and affecting inflammation. An H1R antagonist modulated the expression of the P2 × 7 receptor and suppressed microglial M1-like activation and production of proinflammatory cytokines, including IL-1b and TNF-a, in the hippocampus, thereby alleviating depressive-like behavior in mice (75). These results suggest that histamine plays an important role in neuroinflammation-related diseases, including depression, *via* microglia.

Interestingly, mast cells, which can promote inflammation and allergic reactions *via* the release of histamine, are also present in the brain. Mast cell degranulation and histamine release often promote awareness and positive behavior. For example, compared with wild-type mice, mast cell-deficient mice have increased delta power and reduced food seeking-motivated behavior; however, these mice also have more anxiety and depression than wild-type mice (55).

Recent research on histamine suggests that we need to reassess the role of histamine in the innate and adaptive immune responses in depression. However, the effect of differential expression or activation of histamine receptors on immune cells in depression has not been fully described, and further examination of this effective immunoregulatory network may lead to a better understanding of depression.

### Therapeutic prospects of histamine receptors antagonists for depression

Histamine and its receptors play an important role in the pathophysiology of depression. Recently, some studies have investigated antidepressant drugs related to histamine receptors (Table 2). Rodent studies revealed that acute and chronic treatment with an H3R antagonist can reduce depression-like conditions and that chronic H3R antagonism (*via* ciproxifan) alleviates depression-like conditions in mice *via* the modulation of stress-induced biochemical correlates such as BDNF, corticosterone, NUCB2/nesfatin-1, and CRH in the brain (51, 76, 77). The H3R antagonist clobenpropit reduces the activity of presynaptic H3Rs, increases histamine release, activates hippocampal H1R and H2R, and effectively ameliorates depression-like behavior in FSL mice. Its antidepressant effect can be blocked by H1R and H2R antagonists (H2R-ANTs) (77). In addition, systemic or hippocampal injection of clobenpropit reversed motor and cognitive impairment in a depressed rat model in a manner dependent on the release of histamine and the actions of H1R and H2R (78). The H3R antagonist pitolisant can reduce olanzapine-induced depression-like symptoms in mice (79). The selective and potent H3R inverse agonist JNJ10181457 (JNJ) suppressed microglial chemotaxis and phagocytosis *in vivo* and suppressed microglial activation and improved depression-like behavior *ex vivo* in mice (74). The H3R antagonist/inverse agonist 3,5-dimethyl-isoxazole-4-carboxylic acid (3 h) was effective in the forced swimming test, suggesting its potential therapeutic effect as an antidepressant. After assessment of cardiovascular and neuropsychological/behavioral safety, the compound was widely considered a preclinical drug candidate (80). A quantitative structure–activity relationship (QSAR) study of the serotonin reuptake-inhibitory and H3-antagonistic activity of piperazine and diazepane amide derivatives provided a rational approach for combination treatment with histamine H3 antagonists and serotonin reuptake inhibitors, which may improve not only depressive mood but also cognitive impairment and fatigue in depression (81).

**TABLE 2:** Therapeutic prospect with histamine receptor for depression.

Ligand (Reference)	HR	Animal model	Administered (dose)	Function	Outcome
Ciproxifan (51, 76, 77)	H3R antagonist	Chronic unpredictable stress (CUS) model of depression in C57BL/6 J mice	Intraperitoneal injections (3 mg/kg.; for 3 weeks)	Modulate BDNF, corticosterone, NUCB2/nesfatin-1, and CRH in the brain	Alleviate depression like condition
Clobenpropit (77, 78)	H3R antagonist	Sprague-Dawley Rats or male FSL rats	Subcutaneously injections (5 mg/kg); hippocampal delivery, bilateral guide cannula (10 mM, 15 min);	Increases histamine release, activate hippocampal H1R and H2R	Improve depression-like behavior, reversed the motor and cognitive impairment
JNJ10181457 (JNJ) (74)	H3R inverse agonist	CX3C chemokine receptor 1 (CX3CR1)-green fluorescent protein	Intraperitoneal injections (10 mg/kg.; for 3 days)	Suppress microglial chemotaxis, phagocytosis	Improve depression-like behavior
3 h (3,5-dimethyl-isoxazole-4-carboxylicacid) (80)	H3R antagonist/inverse agonist	Male OF1 mice and male Sprague-Dawley rats	Oral (3 and 10 mg/kg)	Unknown	Improve depression-like behavior
Clemastine (75)	First-generation H1R antagonist	(GFP) mice were injected with LPS (1 mg/kg)	Intraperitoneal injections (10 mg/kg.; for 3 days)	Reduce inflammation	Alleviate stress-related depressive-like behavior

It was suggested that clemastine, a first-generation H1R antagonist, could impressively alleviate stress-related depressive-like behavior in mice by reducing inflammation (75). Interestingly, treatment with a 28-day course of paroxetine increased the mRNA expression of H1Rand histidine decarboxylase in the frontal cortex of mice (82). Although further studies are needed, the literature suggests that histaminergic systems are partially responsible for the pathogenesis of sleep disturbance induced by treatment with selective serotonin reuptake inhibitors (SSRIs) or serotonin-norepinephrine reuptake inhibitors (SNRIs) in rodents (82).

Preclinical studies demonstrated that SSRIs, such as citalopram and paroxetine, and the bioactive lipid mediator oleoylethanolamide (OEA) [but not the tricyclic antidepressant (TCA) imipramine or the selective NA reuptake inhibitor reboxetine] specifically require the brain histamine system to exert their antidepressant-like effects (83, 84). However, a case report indicated that cetirizine, a second-generation H1R antagonist, can induce depression and suicidal behavior, but the symptoms abated with the cessation of cetirizine (85). A meta-analysis of H2R antagonist treatment combined with antipsychotic treatment in patients with schizophrenia showed that H2R antagonist adjuvant therapy did not improve psychotic symptoms but increased the risk of depression (only two comparisons with nizatidine) (86). On the other hand, antihistamines, which are mainly used to treat chronic pruritus, may affect mood and sleep quality. Outpatients treated with cetirizine reported higher depression scores than outpatients treated with desloratadine, levocetirizine, and rupatadine (87).

In summary numerous animal studies have shown that HR is a new target for antidepressant therapy and has the advantage of improving not only emotional symptoms but also cognition. Moreover, a small number of studies have found that H1R antagonists have antidepressant effects. In addition, histamine was found to be involved in the effects of currently available antidepressants (SSRIs). There are several successful clinical treatments: (1) The H1R antagonist, doxepin, can be used for the treatment of insomnia (88), Doxepin can also act as norepinephrine, serotonin reuptake inhibitor and 5-HT2-receptor antagonist to exert antidepressant effect. Its action on histamine is one of its properties beside its effects of norepinephrine and serotonin. (2) The H3R-antagonist/inverse agonist, pitolisant, for the treatment of narcolepsy and obstructive sleep apnea (89–91). But so far there is no clinical study about depression.

This finding was surprising and suggested questions about the potential mechanisms and clinical applications of histamine. Therefore, more research is needed to analyze the role of the histaminergic system in depression and its therapeutic significance.

## Conclusion and future directions

Histamine as a monoamine transmitter is rarely studied for the effect on depression, our review is the first time to illustrate the effect of histaminergic system on depression in order to find the way for the development of new antidepressant drugs. We suggest that histaminergic system may play a pivotal role in the pathogenesis of depression. Histamine and its receptors can regulate the immune response to improve symptoms of depression, and H3R can interact with other transmitters such as NMDA, DA, 5-HT, and MCH which are related to depression. Moreover, H3R and H1R antagonist can alleviate depression in rodent studies, but the clinical potential of these drugs has not been tested. Such research is clearly the next step to open new areas of application.

## Author contributions

GW and HQ conceived the idea of the study and designed the study. All authors analyzed data, wrote the manuscript, discussed the results, and revised the manuscript.
